# Detection and Genetic Characterization of Bovine Torovirus in Uruguay

**DOI:** 10.3390/v16060835

**Published:** 2024-05-24

**Authors:** Matías Castells, María José Benítez-Galeano, Ana Marandino, Rubén Darío Caffarena, María Laura Casaux, Ruben Pérez, Federico Giannitti, Rodney Colina

**Affiliations:** 1 Laboratorio de Virología Molecular, Departamento de Ciencias Biológicas, Centro Universitario Regional (CENUR) Litoral Norte, Universidad de la República, Salto 50000, Uruguay; 2 Unidad de Genómica y Bioinformática, Departamento de Ciencias Biológicas, Centro Universitario Regional (CENUR) Litoral Norte, Universidad de la República, Salto 50000, Uruguay; 3 Sección Genética Evolutiva, Departamento de Biología Animal, Instituto de Biología, Facultad de Ciencias, Universidad de la República, Montevideo 11400, Uruguay; 4 Plataforma de Investigación en Salud Animal, Estación Experimental La Estanzuela, Instituto Nacional de Investigación Agropecuaria (INIA), Colonia 70006, Uruguay; 5 Unidad Académica Salud de Rumiantes, Departamento de Producción y Salud de los Sistemas Productivos, Facultad de Veterinaria, Universidad de la República, Montevideo 13000, Uruguay

**Keywords:** bovine torovirus, cattle, Uruguay, complete genome, genetic diversity, evolution

## Abstract

Bovine torovirus (BToV) is an enteric pathogen that may cause diarrhea in calves and adult cattle, which could result in economic losses due to weight loss and decreased milk production. This study aimed to report the presence, the genetic characterization and the evolution of BToV in calves in Uruguay. BToV was detected in 7.9% (22/278) of fecal samples, being identified in dairy (9.2%, 22/239) but not beef (0.0%, 0/39) calves. BToV was detected in both diarrheic (14%, 6/43) and non-diarrheic (13.2%, 5/38) dairy calves. In addition, BToV was detected in the intestinal contents of 14.9% (7/47) of naturally deceased dairy calves. A complete genome (28,446 nucleotides) was obtained, which was the second outside Asia and the first in Latin America. In addition, partial S gene sequences were obtained to perform evolutionary analyses. Nucleotide and amino acid substitutions within and between outbreaks/farms were observed, alerting the continuous evolution of the virus. Through Bayesian analysis using BEAST, a recent origin (mid-60s) of BToV, possibly in Asia, was estimated, with two introductions into Uruguay from Asia and Europe in 2004 and 2013, respectively. The estimated evolutionary rate was 1.80 × 10^−3^ substitutions/site/year. Our findings emphasize the importance of continued surveillance and genetic characterization for the effective management and understanding of BToV’s global epidemiology and evolution.

## 1. Introduction

Bovine torovirus (BToV) was first detected in 1979 in a farm near Breda, Iowa, United States [1]; hence, it was initially named Breda virus. Later, BToV was allocated into a new family called *Toroviridae* based on the virion’s morphology and structure [2]. Currently, the International Committee on Taxonomy of Viruses classifies BToV in the realm *Riboviria*, order *Nidovirales*, family *Tobaniviridae*, genus *Torovirus*, species *Bovine torovirus* [3].

The name torovirus comes from the Latin *torus* due to the shape of the nucleocapsid, which is elongated and tubular with helical symmetry and can be bent into the shape of a biconcave or kidney-shaped disk. The viral particles have a peplomeric envelope containing the nucleocapsid [2]. The genome comprises a single-stranded RNA molecule of positive polarity and a length of around 28,475 nucleotides [4].

BToV is an enteric pathogen that may cause mild to profuse, potentially life-threatening diarrhea in calves within the first months of life [2,5]. Furthermore, it can affect adult cattle causing watery diarrhea and anorexia, resembling winter dysentery caused by bovine coronavirus (BCoV), which can lead to a decrease in milk production in lactating dairy cows [6]. Clinical disease is mainly mediated by enteritis, but like coronaviruses, it can also infect the respiratory tract [6,7]. Transmission is primarily via the fecal–oral route, but respiratory transmission can also occur [8].

In South America, BToV has been rarely studied with only one report in diarrheic young and adult cattle in Brazil [9]. The member countries of the MERCOSUR Alliance (Argentina, Brazil, Paraguay, and Uruguay) are among the 10 principal beef exporters worldwide [10], and Uruguay and Argentina are also dairy leading exporting countries [11]. In addition, only a few BToV complete genomes have been sequenced, which are almost all from Asia (mainly China). This study aimed to determine the presence of BToV, its genomic characterization and evolution in calves in Uruguay, broadening the current knowledge on this virus’s geographic distribution and genetic diversity.

## 2. Materials and Methods

### 2.1. Samples

A total of 325 samples from a bank stored at −80 °C in the Molecular Virology Laboratory of the CENUR Litoral Norte of the University of the Republic of Uruguay were analyzed, comprising 278 fecal samples from live calves and 47 intestinal contents of naturally deceased calves. These samples had been collected during a four-year period (2015–2018). The selection of the samples was for convenience (availability in the bank of samples). Briefly, fecal and intestinal content samples were diluted 1:10 (*v*/*v*) in phosphate-buffered saline solution and centrifuged at 3000× *g* for 20 min at 4 °C, keeping the supernatants.

### 2.2. Viral RNA Extraction, Reverse Transcription, and Bovine Torovirus Screening by qPCR

Viral RNA was extracted using QIAamp^®^ Viral RNA Mini Kit (QIAGEN^®^, Hilden, Germany), following the manufacturer’s instructions, and tested to detect BToV by reverse transcription (RT) followed by real-time polymerase chain reaction (qPCR). The RT was carried out with RevertAid^®^ Reverse Transcriptase (Thermo Fisher Scientific^®^, Waltham, MA, USA) and random hexamers primers, following the manufacturer’s instructions. The qPCR used for screening targeted the nucleocapsid gene [12]. Briefly, 12.5 µL of SensiFAST™ Probe No-ROX Kit (Bioline^®^, Cincinnati, OH, USA), 5 µL of DEPC-treated DNAse-free water, 1.0 µL of [10 µM] forward primer, 1.0 µL of [10 µM] reverse primer, 0.5 µL of [10 µM] probe, and 5 µL of cDNA were mixed in each tube and analyzed in a Rotor-Gene Q (QIAGEN^®^, Hilden, Germany). Positive and negative controls were included at each step.

### 2.3. Conventional PCR and Sequencing

To obtain sequences for evolutionary analysis, BToV-positive samples were amplified by conventional PCR targeting the S genomic region [13]. Briefly, 5 µL of cDNA, 12.5 µL of MangoMix™ (Bioline^®^, Cincinnati, OH, USA), 4.5 µL of DEPC-treated DNAse-free water, 1.0 µL of dimethyl sulfoxide, 1.0 µL of [10 µM] forward primer 1 (5′-GTGTTAAGTTTGTGCAAAAAT-3′) and 1.0 µL of [10 µM] reverse primer 1 (5′-TGCATGAACTCTATATGGTGT-3′) were mixed. Cycling conditions were as follows: 95 °C for 5 min, 35 cycles consisting of 94 °C for 1 min, 53 °C for 1 min and 72 °C for 1 min, with a final elongation at 72 °C for 10 min. In addition to the samples, positive and negative controls were included. Amplification products of the expected size (722 bp) were visualized in 2% agarose gel and purified with the DNA Clean & Concentrator-5 or Zymoclean Gel DNA Recovery Kit (Zymo Research^®^, Irvine, CA, USA). Purified products were sequenced using Sanger technology in Macrogen, Inc. (Seoul, Republic of Korea).

### 2.4. Characterization of Three BToV Cases

From the 325 samples, and in order to obtain information on BToV evolution, we specifically studied three BToV-associated cases in three farms (namely A, B and C) with at least two BToV-positive calves. Farm A consisted of samples collected in July 2015 from 6 calves, with 4 of them (A1, A2, A3 and A4) being positive for BToV; farm B consisted of samples collected in September 2015 from 8 calves, with 2 of them (B1 and B2) positive for BToV; and farm C consisted of samples collected in April 2017 from 5 calves, with 2 of them (C1 and C2) positive for BToV.

### 2.5. Complete Genome

In addition to the analyzed samples, a complete genome was obtained during a next-generation sequencing reaction from a fecal sample obtained in May 2019 from a Holstein dairy calf of approximately 10 days of age from San José, Uruguay, that died after having neonatal calf diarrhea (NCD) syndrome.

The fecal suspension was filtered using 0.45 µm membrane filters. Viral RNA was extracted with Quick-RNATM MiniPrep kit (Zymo Research^®^, Irvine, CA, USA), employing the DNAse treatment suggested by the manufacturer, and converted to double-stranded cDNA with a Maxima H Minus kit using random primers (Thermo Fisher Scientific^®^, Waltham, MA, USA). The library preparation was performed using the Nextera™ DNA Flex Library Preparation kit (Illumina^®^, Sao Paulo, Brazil). Purified libraries were quantified using the Qubit system (Thermo Fisher Scientific^®^, Waltham, MA, USA) and sequenced on the Illumina MiniSeq (Illumina^®^, Sao Paulo, Brazil) genomic platform of the Universidad de la República with the MiniSeq TM Mid Output Reagent Cartridge (300 cycles, paired-end reads).

Raw data in fastq format were quality-filtered and trimmed using CLC Genomics Workbench 24.0 (QIAGEN^®^, Hilden, Germany). Adapter sequences were removed, and reads with quality scores higher than 30, with up to two ambiguous nucleotides and longer than 120 bp, were retained. After trimming, reads were de novo assembled, and contigs were subjected to BLAST [14]. Contigs that significantly aligned (percentage of identity >95%) with Torovirus genomes were mapped with the CLC mapper to the bovine torovirus strain SC-1 Sichuan/2018 (MN073058) reference sequence for further analysis. 

After mapping, five regions were missed from the entire genome; thus, five conventional PCRs targeting the five missing regions were designed to obtain the entire genome. Briefly, 4 µL of cDNA, 10 µL of MangoMix™ (Bioline^®^, Cincinnati, OH, USA), 3.6 µL of DEPC-treated DNAse-free water, 0.8 µL of dimethyl sulfoxide, and 0.8 µL of [10 µM] forward primer and 1.0 µL of [10 µM] reverse primer were mixed in 0.2 mL microtubes; the primers sequences are detailed in Table 1. PCR conditions were as follows: 95 °C for 5 min, followed by 40 cycles of 94 °C for 1 min, 45 °C for 1 min, and 72 °C for 1 min, ending with a final elongation step of 10 min at 72 °C. Amplification products were visualized in 2% agarose gel and purified with the DNA Clean & Concentrator-5 (Zymo Research^®^, Irvine, CA, USA). Purified products were sequenced using Sanger technology in Macrogen, Inc. (Seoul, Republic of Korea).

### 2.6. Sequence Analyses

The partial S gene sequences obtained in this study were deposited in GenBank with accession numbers MW002687-95 and PP445198-200, and the complete genome sequence was deposited with accession number PP445201. All the BToV and related sequences corresponding to the S fragment analyzed here (positions 21,020 to 21,583 by reference sequence BToV/URY/Chiriru5413/2019, accession number PP445201) with information of country and date of collection were downloaded from GenBank. In addition, all the available torovirus genomes were downloaded from GenBank. The multiple sequence alignment of the partial S sequences was obtained using MEGA11 [15], and the multiple sequence alignment of complete genomes was obtained using Clustal Omega [16]. The best model of nucleotide substitution and the maximum likelihood trees were obtained with W-IQ-TREE [17]. Branch support was assessed with 1000 replicas of the SH-aLRT test [18].

A phylogeographic study was conducted to investigate the evolutionary history and geographic dispersion of BToV and delve deeper into the evolution of the virus. Only sequences corresponding to the partial S region sequenced in our study and with sampling date information were included. The temporal structure of the dataset was evaluated using TempEst [19]. The best-fit substitution model determined previously (TN+I+G4) was used in the analysis implemented in the BEAST v1.8.4 package [20]. A strict molecular clock and a Bayesian skyline coalescent model were used as priors. To obtain the phylogeographic information, the country of detection was used as a trait. A Markov chain Monte Carlo (MCMC) length of 100 million generations was run, obtaining 10,000 parameter samples. The effective sample size (ESS) was evaluated in Tracer v1.6.0, and ESS values higher than 200 for all parameters were accepted. A maximum clade credibility tree was obtained using TreeAnnotator software from the BEAST v.1.8.4 package and visualized in FigTree v1.4.3. A Bayesian Skyline plot was generated using Tracer v1.6.0.

### 2.7. Statistical Analysis

The detection frequencies were compared between live and deceased, diarrheic and non-diarrheic, beef and dairy calves, and calves born from dams vaccinated and non-vaccinated. Categorical data were evaluated through Chi-square except in cases with values lower than 5 in which Fisher exact tests was used; differences were considered statistically significant if the obtained *p*-value was <0.05.

## 3. Results and Discussion

The global prevalence (including all the 325 samples) was 8.9% (29/325). The frequency of BToV detection in fecal samples was 7.9% (22/278, 95% CI: 5.03–11.74%). BToV has been detected in several countries with a frequency ranging from 1.1% to 43.2% [21,22]. In Brazil, a country bordering Uruguay, the observed frequency was similar (6.25%, 5/80) to that reported in our study [9]. 

This is the first study of BToV, including samples of dairy and beef calves from the same country. Notable, BToV was only detected in dairy (9.2%, 22/239, 95% CI: 5.86–13.6%) but not beef (0.0%, 0/39, one-sided 97.5% CI: 0.0–9.03%) calves; this difference was not statistically significant (*p* = 0.052). A limitation of this study was the overrepresentation of dairy calves compared to beef calves. The detection of BToV suggests the circulation of a virus that potentially has a negative effect in cattle production in one of the major exporters of beef and dairy products worldwide [10,11].

We did not find differences in the proportion of BToV detection between diarrheic (14%, 6/43) and non-diarrheic (13.2%, 5/38) dairy calves (*p* = 0.93). Unfortunately, since this was a convenience sampling study, some metadata were unavailable or unclear for several samples. Despite other studies having demonstrated that BToV is more often detected in diarrheic than non-diarrheic calves [13,21,23,24], this was not observed in Uruguay. Nevertheless, it was not the aim of this study to identify an eventual causal association between BToV infection and disease, but this result indicates that BToV is shed both by symptomatic and asymptomatic calves, as is the case of other enteropathogenic viruses of calves (i.e., coronavirus and group A rotavirus) [25,26,27]. In Uruguay, the annual dairy calf mortality risk between birth and weaning (75 days of age) is high (15.2%) [28], which not only causes important economic losses but also is concerning from the animal welfare standpoint. As BToV has the potential to cause disease in susceptible animals, its detection in both symptomatic and asymptomatic animals indicates that management practices on farms should consider the potential role that asymptomatic animals may play in viral spread. The NCD syndrome has been identified as an important contributor to dairy calf mortality [28,29], and while several viral, bacterial, and protozoal enteric pathogens have been investigated in NCDs of dairy calves in the country [25,27,29,30,31,32], BToV has not. Further studies are needed to assess the clinical and economic impact of BToV in Uruguay in particular and South America and other livestock producing countries in general.

The fecal samples analyzed in this study were collected over a four-year period (2015–2018); BToV was detected in samples collected in 2015 (25.9%, 7/27), 2016 (12.6%, 11/87), and 2017 (3.3%, 4/122), while it was not detected in samples from 2018 (0.0%, 0/42).

Regarding the geographic location of the live calves, we analyzed samples from 8 of the 19 departments Uruguay is geographically subdivided into. The geographic information was available for 16 (72.7%) of the 22 BToV-positive live calves, which were mainly from Colonia (93.8%, 15/16), with a single exception from Soriano (6.2%, 1/16). BToV was not detected in the other 6 departments: Cerro Largo (0/6), Florida (0/8), Río Negro (0/9), Rocha (0/17), San José (0/36), and Tacuarembó (0/8), although geographic information for 6 of the 22 BToV-positive calves was unavailable. The frequency of BToV detection was 34.9% (15/43) in Colonia and 9.1% (1/11) in Soriano. At the herd level, 4 of 5 (80%) herds from Colonia had at least one BToV-positive calf. Surveillance should be maintained over time to detect possible factors influencing BToV circulation.

The frequency of BToV detection in naturally deceased calves was also analyzed. Although BToV was detected with a higher frequency in deceased (14.9%, 7/47) than live (9.2%, 22/239) dairy calves, the difference was not statistically significant (*p* = 0.12). Despite BToV having been recognized as a causative agent of diarrhea, the epidemiological data for this viral infection are limited. Clinical signs are similar to those caused by BCoV infections, so it has been suggested that BToV may have been misdiagnosed as BCoV infection by clinical investigations [6]. In addition to the clinical similarities between both viruses, in Uruguay, BToV and BCoV showed epidemiological similarities too, with similar frequencies of detection in live (7.9% and 7.8%, respectively) and deceased (10.0% and 14.9%, respectively) calves, and both were detected in similar proportions in diarrheic and non-diarrheic calves [25]. In addition, a higher frequency of infection with either virus was observed in 4-week-old calves, which was followed by calves of 3, 1, and 2 weeks of age, respectively [25]. On the other hand, some differences were observed; while BCoV was detected in beef cattle, BToV was not, and BCoV was geographically widespread, whereas BToV was detected only in the southwestern region of the country (Colonia and Soriano). Finally, something noteworthy was that in this study, the detection of BToV in calves born from NCD-vaccinated dams (5.1%, 3/59) was significantly lower (*p* < 0.001) than in those born from non-vaccinated dams (42.3%, 11/26), as previously observed for BCoV [25]. Even though there are no available vaccines against BToV, this observation could be related to better management and husbandry practices in farms that vaccinate than those that do not. Additional studies should be performed to understand and contribute with data to prevent and control BToV transmission.

Due to the multifactorial nature of the NCD syndrome, it is difficult to determine the role of each agent at the herd and animal levels. In addition to viruses, other pathogens such as bacteria and/or protozoa are usually involved, as well as other calves’ factors, such as their immune status, age, nutritional status, and passive immunity intake, among others [33]. However, the prevalence of BToV in Uruguay suggests that it may play a role in cattle health and disease. 

The S gene, specifically the fragment sequenced in this study, is relatively abundant in GenBank. This gene codifies the spike protein, which is a surface protein that determines the antigenicity [13]. Thus far, two serotypes have been described, and both caused diarrhea in gnotobiotic calves [34]. In addition, the complete genome of serotype 1 has been obtained [4], but no sequence was available for serotype 2, which may indicate that it is not widely dispersed and/or not prevalent. 

We studied the evolution of BToV in real time through three farms with at least two BToV-positive calves. Interestingly, within farm A, we observed sequences encompassing fragment S with three different patterns. The strains detected in calves A1 and A2 were identical at the nucleotide and amino acid levels. Nevertheless, comparing with strains A1/A2, strain A3 (BToV/URY/LVMS669/2015) had a nucleotide substitution A634T that led to amino acid substitution T212S, and strain A4 (BToV/URY/LVMS665/2015) showed the nucleotide substitution A715C, which also changed the amino acid T239P. Both strains within farm B were identical between them, and both strains within farm C were also identical between them. Between farms, strains A1/A2 and strains B1/B2 were similar, with only one nucleotide difference, that led to an amino acid substitution, with glutamine in the strains of farm A and glutamic acid for strains of farm B. Interestingly, in the region of the spike gene studied (which had a length of 650 nucleotides), strains C1/C2 presented 49 and 48 nucleotide differences with strains A1/A2 and B1/B2, respectively. These findings suggest ongoing genetic variation in BToV. This variation is observed between different farms in the same year and within a single farm. The virus may also undergo antigenic drift, similar to what has been observed in BCoV, where a single amino acid change in the spike protein made the virus resistant to neutralization [35]. Although the possibility of an error during the RT-PCR and sequencing cannot be definitely ruled out, both DNA strands were sequenced to obtain the consensus, minimizing the chances of error.

Our evolutionary study used a partial region of the spike gene to estimate significant viral parameters, including the evolutionary rate, the most recent common ancestor, the geographical origin, and the dispersal pattern. The dataset comprised all BToV sequences available in GenBank with information on the date and country of detection and closely related sequences from toroviruses found in a goat and a Tasmanian devil. The average evolutionary rate was estimated to be 1.80 × 10^−3^ substitutions/site/year (s/s/y, 95%HPD 1.35–2.30 × 10^−3^ s/s/y). This rate falls within the typically observed range for RNA viruses [36] and is consistent with the rate reported for the M genomic region of torovirus [37].

**Figure 1:**
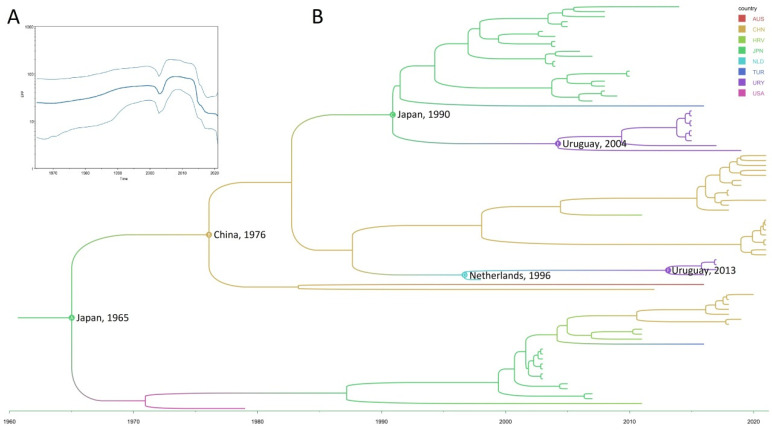
Bayesian MCMC analysis. (**A**) Bayesian skyline. The effective population size through the time is shown. (**B**) Maximum clade credibility tree. The date and most probable country of origin are shown in key nodes.

Regarding the viral population analyzed through the Bayesian Skyline (Figure 1A), a slight increase in cases was observed between 2005 and 2015, which was followed by a decline from 2015 to the present. This trend is consistent with the drop in the detection frequency in Uruguay between 2015 and 2018, which was the sampling period included in this study.

The most recent common ancestor was dated back to 1965 (95%HPD 1954–1974) (Figure 1B), indicating a recent origin of BToV, which was most probably in Asia (51%). Japan (28%) and China (23%) are the most probable countries of emergence within Asia, despite the first detection being reported in the United States in 1979 [1], 15 years after the date of emergence estimated herein. Nonetheless, the possibility of the virus emerging in another region or country cannot be ruled out, as this study was conducted only with the limited set of available sequences. Furthermore, it is worth mentioning that a probability of 26% exists that BToV could have emerged in the United States.

The most probable geographical dispersion of the virus from its emergence until reaching Uruguay is as follows: as mentioned, it likely originated in Japan (28%), passing then through China, where it diversified into two distinct groups with different paths. One of these groups returned to Japan (70% probability) before reaching Uruguay in 2004, while the other passed through the Netherlands (74% probability) before arriving in Uruguay in 2013. Recently, two introductions of BCoV to Uruguay were described [25], both in 2013, similar to the latest introduction of BToV observed in this study, showing a similar pattern between both related viruses.

Finally, we obtained a complete genome sequence of BToV using NGS and Sanger technologies. After trimming and quality filtering Illumina raw data, about 8576 reads with an average length of 139 bp were assembled in seven contigs of variable length, the longest with 19,760 bp in length. Overall, the average coverage of the almost complete genome obtained from these contigs was around 43x. Contigs were mapped to strain BToV SC-1 Sichuan/2018 (MN073058), and 94% of the genome was covered. The remaining gaps to complete the genome, about 1718 bp, were filled with PCRs and Sanger sequencing. There are few BToV complete genomes in the GenBank database with only 20 complete genomes available as of March 2024 and four additional but unverified genomes. From the 20 verified complete genomes, one is from the USA, the Breda 1 strain from 1979 (the bovine torovirus reference sequence), and 19 are from Asia, with 17 from China and 2 from Japan. Thus, ours represents the first complete genome from Latin America. This expands the geographical availability of genomes, which is essential for virus evolution studies and its geographical dispersion.

The full genome contains the five common ORFs of other toroviruses: ORF1a codes the replicase (nucleotides 915–14,168), the S gene codes the spike, a structural glycoprotein (nucleotides 20,947–25,701), the M gene codes the membrane structural glycoprotein (nucleotides 25,730–26,431), the HE gene codes the hemagglutinin esterase (nucleotides 26,449–27,708), and the N gene codes the nucleocapsid phosphoprotein (nucleotides 27,751–28,242). At the nucleotide level, the Uruguayan genome shared the highest percentage of identity (96.56%) with the strain BToV SC-1 Sichuan/2018 (MN073058) from China. The ORF1a gene had 96.91% shared identity with the strain BToV14/2021/CHN (ON337876) from China, the S gene had 95.94% shared identity with the strain B145 (AJ575373) from the Netherlands, the M and HE genes had 97.86% shared identity with the strain BToV Kagoshima/2014 (LC088095) from Japan, and the N gene had 97.97% shared identity with the strain B156 (AJ575385) from the Netherlands. At the amino acid level, in the replicase, it shared the highest percentage of identity (96.99%) with the strain BToV SC-1 Sichuan/2018 (QHN70901) from China, in the spike, it had 97.66% shared identity with the strain B145 (CAE01339) from the Netherlands, in the membrane protein, it was identical to the strain K-683 (BAF33331) from Japan, in the hemagglutinin esterase, it had 96.90% shared identity with the strains BToV Kagoshima/2014 (BAU21414), BTOV_China/BToV cattle_SC01/2020 (UOF75532) and BToV-HT2-TUR (AYU75163) from Japan, China and Turkey, respectively, and in the nucleocapsid, it had 96.39% shared identity with the strains BToV10/2021/CHN (WDE20496) and BToV-HT2-TUR (AYU75164) from China and Turkey, respectively.

**Figure 2:**
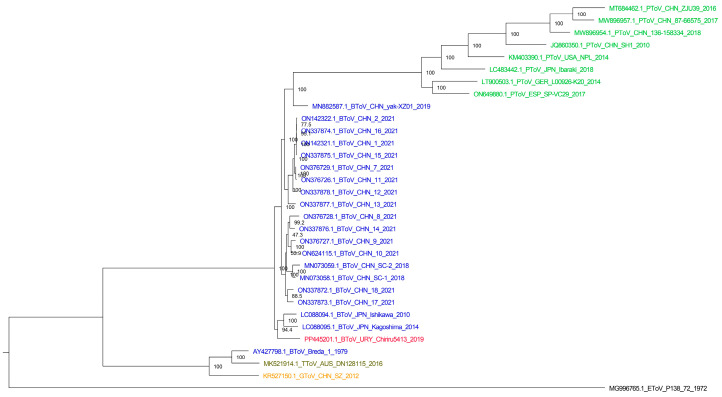
Maximum likelihood tree with complete genomes. Bovine torovirus sequences are in blue font (the Uruguayan sequence described in this study is in red font), porcine torovirus sequences names are in green, Tasmanian devil torovirus sequence name in brown, goat torovirus sequence name in yellow and equine torovirus sequence name in black. SH-aLRT values are shown at each node.

The phylogenetic analysis using complete genomes revealed intriguing findings (Figure 2). The Uruguayan sequence clustered with two Japanese sequences, while the Chinese sequences formed another distinct group. Remarkably, the Breda 1 sequence clustered alongside torovirus sequences detected in a Tasmanian devil and a goat, suggesting a potential interspecies transmission event between cattle and other hosts or vice versa. Subsequently, the strains might have diversified and adapted to bovine hosts. Additionally, a sequence detected in yaks grouped with porcine torovirus sequences, implying a possible origin of porcine torovirus from yak (bovine torovirus) through interspecies transmission. Furthermore, the equine torovirus sequence clustered separately from other torovirus sequences available in the database. As previously mentioned, the scarce availability of complete genomes limits this kind of analysis, which underscores the significance of this study. Obtaining a complete genome from South America, where these data were missing up to date, strengthens the understanding of torovirus diversity and evolution.

## Figures and Tables

**Table 1:** Primers used to complete the gaps in the genome obtained by the next-generation sequence reaction.

Primer Name	Sequence (5′–3′)	Position	Fragment Length
BoToV_PF-1	AGTTCAAATGGACTACCGGGTC	1028–1049	402
BoToV_PR-1	TTAGTTTCAGCAAGAGCCGGG	1409–1429	
BoToV_PF-2	GATGTTTTGGTCAACAACCC	3408–3429	255
BoToV_PR-2	CCTACCAACTGGTTTAACG	3644–3662	
BoToV_PF-3	GGACAGAAGAGTAACAGAGC	24,211–24,230	805
BoToV_PR-3	TGATGGATGAAATGTCAGGC	24,996–25,015	
BoToV_PF-4	CCTTTACTGGTTATTGGGCC	25,867–25,886	599
BoToV_PR-4	GACTCATAGGTGAATAAGGG	26,446–26,465	
BoToV_PF-5	TACAATGCATCTACTGTTGG	27,083–27,102	780
BoToV_PR-5	CCTATTAGCACGTTGTTGGG	27,843–27,862	

## Data Availability

The raw data supporting the conclusions of this article will be made available by the authors on request.
